# Transcriptional landscape of human microglia implicates age, sex, and *APOE*‐related immunometabolic pathway perturbations

**DOI:** 10.1111/acel.13606

**Published:** 2022-04-06

**Authors:** Tulsi Patel, Troy P. Carnwath, Xue Wang, Mariet Allen, Sarah J. Lincoln, Laura J. Lewis‐Tuffin, Zachary S. Quicksall, Shu Lin, Frederick Q. Tutor‐New, Charlotte C.G. Ho, Yuhao Min, Kimberly G. Malphrus, Thuy T. Nguyen, Elizabeth Martin, Cesar A. Garcia, Rawan M. Alkharboosh, Sanjeet Grewal, Kaisorn Chaichana, Robert Wharen, Hugo Guerrero‐Cazares, Alfredo Quinones‐Hinojosa, Nilüfer Ertekin‐Taner

**Affiliations:** ^1^ Department of Neuroscience Mayo Clinic Jacksonville Florida USA; ^2^ Department of Quantitative Health Sciences Mayo Clinic Jacksonville Florida USA; ^3^ Department of Cancer Biology Mayo Clinic Jacksonville Florida USA; ^4^ Department of Neurosurgery Mayo Clinic Jacksonville Florida USA; ^5^ Neuroscience Graduate Program Mayo Clinic Graduate School of Biomedical Sciences Mayo Clinic Rochester Minnesota USA; ^6^ Regenerative Sciences Training Program Center for Regenerative Medicine Mayo Clinic Rochester Minnesota USA; ^7^ Department of Neurology Mayo Clinic Jacksonville Florida USA

**Keywords:** *APOE*, lipid metabolism, microglia, neurodegeneration, single cell, transcriptomics

## Abstract

Microglia have fundamental roles in health and disease; however, effects of age, sex, and genetic factors on human microglia have not been fully explored. We applied bulk and single‐cell approaches to comprehensively characterize human microglia transcriptomes and their associations with age, sex, and *APOE*. We identified a novel microglial signature, characterized its expression in bulk tissue and single‐cell microglia transcriptomes. We discovered microglial co‐expression network modules associated with age, sex, and *APOE*‐ε4 that are enriched for lipid and carbohydrate metabolism genes. Integrated analyses of modules with single‐cell transcriptomes revealed significant overlap between age‐associated module genes and both pro‐inflammatory and disease‐associated microglial clusters. These modules and clusters harbor known neurodegenerative disease genes including *APOE*, *PLCG2*, and *BIN1*. Meta‐analyses with published bulk and single‐cell microglial datasets further supported our findings. Thus, these data represent a well‐characterized human microglial transcriptome resource and highlight age, sex, and *APOE*‐related microglial immunometabolism perturbations with potential relevance in neurodegeneration.

## Graphical abstract

Using bulk and single‐cell approaches, we identified microglial subclusters enriched for different biological functions. Activated microglia were more abundant with aging and lipid localization genes were upregulated. In *APOE‐ε4* carriers, homeostatic microglia were reduced along with downregulation of carbohydrate metabolism genes, whereas cholesterol absorption genes were unregulated in macrophages and activated microglia.

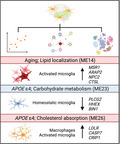

**Abbreviations**
AD: Alzheimer’s disease
AMP‐AD: Accelerating Medicines Partnership Alzheimer’s Disease
CNS: central nervous system
CQN: Conditional Quantile Normalization
DAM: disease‐associated microglia
DNET: dysembryoplastic neuroepithelial tumor
FACS: Fluorescence‐activated cell sorting
GBM: glioblastoma multiforme
GEM: gel beads‐in‐emulsion
ID: identifier
INF: Infinity
MACS: Magnetic‐activated cell sorting
MSBB: Mount Sinai brain bank
MM: Module membership
PCA: Principal components analysis
RPKM: reads per kilo bases per million
QC: quality control
ROS‐MAP: Rush University Religious Orders Study and Memory and Aging Project
scRNAseq: single cell RNA sequencing
snRNAseq: single nuclei RNA sequencing
UMAP: Uniform Manifold Approximation and Projection
UMI: Unique Molecular Identifier
WGCNA: weighted gene co‐expression network analysis

## 1 INTRODUCTION

Microglia are the resident macrophages of the central nervous system (CNS), responsible for clearance of cellular debris and pathological protein aggregates. In the healthy brain, they exist in a homeostatic state and can be induced to a reactive state in response to changes in the CNS microenvironment, such as inflammation and neuronal damage (Masuda et al., 2020). They are fundamental to maintaining brain homeostasis during development, aging, and disease; therefore, microglial dysfunction could ultimately lead to neurodegeneration (Li & Barres, 2018). Microglia are integral to the pathophysiology of neurodegenerative diseases, including Alzheimer's disease (AD) and multiple sclerosis, with chronic inflammation implicated as a contributing factor (Hickman et al., 2018; Keren‐Shaul et al., 2017; Krasemann et al., 2017).

Fresh human brain tissue studies are imperative to the characterization of the microglial transcriptome in health and disease; however, accessibility is limited. Although single nuclei studies using frozen tissue provide an easier alternative, recent studies have demonstrated limitations in detecting substantial populations of less abundant cell types (Del‐Aguila et al., 2019; Mathys et al., 2019). Additionally, it was recently reported that many microglial activation genes are expressed in the cytosol and therefore are likely to be missed by single nuclei RNA sequencing (snRNAseq; Thrupp et al., 2020). Recent single‐cell studies aiming to characterize microglial gene expression using fresh tissue have highlighted the heterogeneity in microglial phenotypes (Masuda et al., 2019; Olah et al., 2020; Sankowski et al., 2019). This has revealed that phenotypic changes are not binary but rather a spectrum of states in which microglia can simultaneously co‐exist during transition from homeostatic to more reactive states. Additionally, these different subsets could have specialized functions in brain homeostasis and dysfunction. Thus, it is increasingly important to characterize these heterogeneous subpopulations to understand their roles in health and disease. This could also help facilitate the design of novel therapeutic approaches to target‐specific subpopulations of cells and modulate their activity (Li & Barres, 2018).

Obtaining fresh human tissue from neurosurgeries allows us to study the mechanisms of microglial function in living cells. Unfortunately, this tissue is usually excised from surgical procedures for tumor resection or relieving temporal lobe epilepsy, rendering it difficult to distinguish between normal and disease‐affected tissue. Darmanis et al. (2017) investigated the effect of GBM tumors on CNS cell types and surrounding tissue, revealing that myeloid cells are greatly affected by the tumor microenvironment. They found that peri‐tumor myeloid populations were primarily pro‐inflammatory microglia compared to macrophages within the tumor core (Darmanis et al., 2017). In temporal lobe epilepsy, two distinct microglial phenotypes have been identified with microglia present in sclerotic areas with few neurons expressing markers of activation, including anti‐inflammatory cytokine IL10 (Kinoshita & Koyama, 2021; Morin‐Brureau et al., 2018). The other phenotype occurs transiently following a seizure, with secretion of interleukins CXCL8 and IL1B mediated by the NLRP3 inflammasome (Morin‐Brureau et al., 2018).

Microglial expression has also been shown to be affected by aging (Galatro et al., 2017; Olah et al., 2018); however, few studies have investigated the effects of sex and genetic factors on human microglia. Sex differences in microglia have been previously reported in mice, with females being predisposed to harboring more activated microglia than males (Frigerio et al., 2019; Nelson et al., 2017; Stephen et al., 2019). *APOE*, a lipoprotein of which the ε4 allele (*APOE*‐ε4) is a major risk factor for AD and also implicated in other neurodegenerative diseases (Yamazaki et al., 2019]), is upregulated in disease‐associated microglia (DAM) in mice and humans, but downregulated in astrocyte and oligodendrocyte subpopulations (Grubman et al., 2019; Hammond et al., 2019; Keren‐Shaul et al., 2017; Mathys et al., 2019). In microglia and neurons, *APOE* interacts with LDL receptors to facilitate endocytosis of cholesterol and phospholipids and modulate lipid homeostasis in the brain (Gamache et al., 2020). Such studies provide growing support for cell type‐specific functions of *APOE*; however, its effects on microglia remain to be fully elucidated. Thereby, identifying age, sex, and *APOE*‐associated pathways in microglia will provide greater insight into the functions of specific microglial subsets in relation to these risk factors. Interindividual variability and diversity in functional states makes targeting specific microglial subsets in disease challenging for modulating these cells (Li & Barres, 2018). Identifying the mechanisms regulating microglial homeostasis and activation can allow us to manipulate these cells for therapeutic purposes.

In this study, we leveraged both bulk and single‐cell approaches to provide a comprehensive characterization of the adult human microglial transcriptome. We obtained fresh intraoperative neurosurgical brain tissue and isolated an enriched population of microglial cells to investigate transcriptional changes associated with age, sex, and *APOE*‐ε4 in bulk microglia and further explored these in single microglial cells. Our findings support age‐, sex‐, and *APOE*‐related microglial transcriptome changes involving lipid and carbohydrate metabolic pathways and implicate microglial immunometabolism perturbations relevant to neurodegenerative diseases.

## 2 RESULTS

**FIGURE 1:**
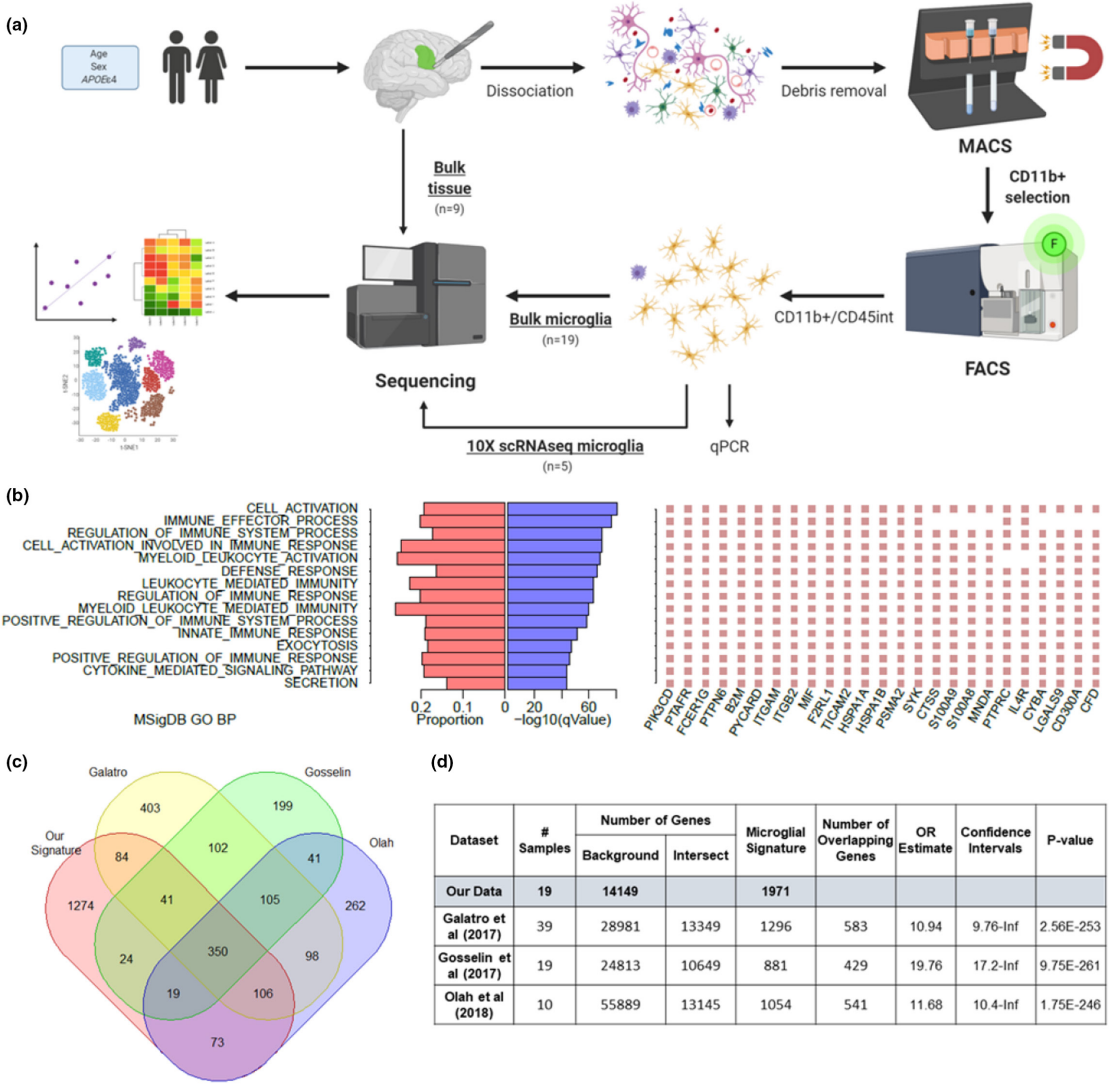
Characterization of our core human microglial signature. (a) Schematic illustrating our experimental approach for isolating microglial populations from fresh brain tissue and data analyses. [Created with BioRender.com] (b) MSigDB GO terms enriched in our microglial signature genes and top 25 genes for each. (c) Venn diagram showing number of overlapping genes between our microglial signature and those previously reported from Galatro et al. (2017), Gosselin et al. (2017) and Olah et al. (2018). (d) Hypergeometric tests of overrepresentation showing overlap with the published signatures

To uncover microglial transcriptional profiles and their associations with age, sex, and *APOE*, we performed microglial cell type‐specific and single‐cell RNA sequencing (scRNAseq) studies in fresh human brain tissue. We obtained neurosurgical tissue unaffected by the primary disease process from 19 human donors (Figure S1). Microglia were isolated by CD11b^+^ microbead selection followed by FACS sorting of cells expressing the CD11b^+^/CD45^intermediate^ microglial signature to acquire a more purified population. These samples underwent bulk microglia RNAseq, with subsets of these and additional samples also undergoing 10x scRNAseq (*n* = 5) and bulk tissue RNAseq (*n* = 9; Figure 1a; Table 1). Validation of sorted microglia using qPCR showed the expected *CD11b* ^+^/*CD45* ^intermediate^/*P2RY12* ^+^ microglial signature (Li & Barres, 2018) with no expression of other cell type markers, indicating that we isolated a highly enriched microglial population (Figure S2).

**TABLE 1:** Demographics table of all samples included in the study and the associated demographics

ID	Group	Sex	APOE genotype	Age	Ethnicity	Brain hemisphere	Brain region	Diagnosis	Bulk microglia	Bulk tissue	Single‐cell microglia	Surgical approach
11	F, *APOE*e4‐neg	F	33	19	White	Right	Temporal	Oligodendroglioma	X			Right temporal craniotomy for parahippocampal/brainstem low‐grade glioma
13	F, *APOE*e4‐neg	F	33	35	Black or African American	Left	Temporal lobe	Epilepsy	X			Left anterior temporal lobectomy
1	F, *APOE*e4‐neg	F	33	37	Hispanic or Latino	Right	Cerebellum	Metastatic carcinoma	X			Right craniotomy for tumor
23	F, *APOE*e4‐neg	F	33	37	White	Right	Frontal	Oligodendroglioma		X		Right awake craniotomy for perirolandic low‐grade glioma
20	F, *APOE*e4‐neg	F	23	45	White	Left	Temporal	GBM	X			Left awake craniotomy with cortical and subcortical mapping for 2 h for insular high‐grade glioma
10	F, *APOE*e4‐neg	F	33	62	White	Right	Temporal insular	GBM	X	X	X	Right temporal craniotomy for temporal insular high‐grade glioma with 5‐ALA
24	F, *APOE*e4‐neg	F	33	64	Hispanic or Latino	Left	Temporal	GBM		X		Left temporal craniotomy for high‐grade glioma
12	F, *APOE*e4‐neg	F	33	65	White	Left	Temporal	Astrocytoma	X			Left temporal craniotomy for low‐grade glioma
21	F, *APOE*e4‐neg	F	33	66	White	Left	Temporal	Meningioma		X		Left temporal craniotomy for tentorial meningioma
4	F, *APOE*e4‐neg	F	33	71	White	Right	Temporal lobe	Astrocytoma	X			Right temporal craniotomy for glioma
17	F, *APOE*e4‐pos	F	**24**	37	White	Left	Insula	Astrocytoma	X			Left awake craniotomy with cortical and subcortical mapping for insular glioma
14	F, *APOE*e4‐pos	F	**34**	47	White	Right	Parietal cortex	GBM	X	X		Right parietal craniotomy for parieto‐occipital high‐grade glioma with brain path tubular retractor
8	F, *APOE*e4‐pos	F	**34**	62	White	Right	Temporal/occipital	GBM	X			Right temporal craniotomy for high‐grade glioma
15	F, *APOE*e4‐pos	F	**34**	63	White	Right	Temporal	Metastatic carcinoma	X	X	X	Right Temporal craniotomy for tumor resection
9	M, *APOE*e4‐neg	**M**	33	19	African American	Left	Temporal	DNET	X			Left temporal craniotomy for resection of mesiotemporal glioma with BrainPath retractor
2	M, *APOE*e4‐neg	**M**	23	25	White	Left	Mesial temporal lobe	Epilepsy	X			left anterior temporal lobectomy
5	M, *APOE*e4‐neg	**M**	33	27	Hispanic or Latino	Right	Mesial temporal cortex	Epilepsy	X		X	Right temporal lobectomy
6	M, *APOE*e4‐neg	**M**	33	27	Hispanic or Latino	Right	Anterior temporal cortex	Epilepsy			X	Right temporal lobectomy
26	M, *APOE*e4‐neg	**M**	33	27	White	Left	Temporal	Astrocytoma			X	Left awake craniotomy for tumor resection, non‐IMRI
19	M, *APOE*e4‐neg	**M**	33	31	Hispanic or Latino	Right	Frontal	Oligodendroglioma	X		X	Right awake craniotomy with brain mapping and electrocorticography for tumor resection
3	M, *APOE*e4‐neg	**M**	33	34	White	Right	Temporal insular	Oligodendroglioma	X			Right awake temporal craniotomy for temporal insular glioma
7	M, *APOE*e4‐neg	**M**	23	55	White	Right	Temporal lobe	GBM	X			Right temporal craniotomy for high‐grade glioma
16	M, *APOE*e4‐neg	**M**	33	59	White	Right	Frontal	Oligodendroglioma	X			Right awake craniotomy with cortical and subcortical mapping for low‐grade glioma
18	M, *APOE*e4‐neg	**M**	33	66	Middle Eastern	Left	Frontal	GBM	X	X		Left frontal craniotomy for tumor resection
22	M, *APOE*e4‐pos	**M**	**34**	67	White	Left	Frontal	Meningioma		X		Left pterional craniotomy for tumor resection
25	M, *APOE*e4‐NA	**M**	NA	63	White	Left	Frontal	GBM		X		Left frontal craniotomy for opercular high‐grade glioma

Note

Samples were either included for bulk microglial RNAseq, bulk tissue RNAseq, 10× Genomics single cell RNAseq or on multiple platforms. Samples 5 and 6 were from two brain regions of the same person.

M = male, F = female, GBM = glioblastoma multiforme, DNET = dysembryoplastic neuroepithelial tumor, males and APOE‐ε4 carriers are shown in bold.

### 2.1 Identification of a core human microglial transcriptional signature

To define a core human microglial signature, we calculated log_2_ fold change and *q*‐values of differential expression for each gene between bulk microglia RNAseq data in our study and bulk brain RNAseq data from 7 AMP‐AD datasets provided by Mayo Clinic (Allen et al., 2016]), Mount Sinai Brain Bank (Wang et al., 2018), and Rush University Religious Orders Study and Memory and Aging Project (ROSMAP; De Jager et al., 2018) representing 6 brain regions from 515 human samples. Using a cutoff of 4‐fold greater expression in our bulk microglia and a *q*‐value threshold of 0.05, we identified 1971 genes (Table S1–S6). These genes were expressed at significantly greater levels in our bulk microglial transcriptome data in comparison with each of the bulk brain transcriptome datasets. Therefore, we considered these 1971 genes as the core microglial signature in our dataset. This signature comprises several known marker genes, with 12.7% of the genes being BRETIGEA (McKenzie et al., 2018) microglial genes, suggesting that it also likely harbors novel microglial markers of interest (Table S7). GO enrichment using MSigDB showed that this signature was enriched for genes involved in immune‐related and inflammatory response pathways as would be expected, and leukocyte‐mediated immunity (Figure 1b).

To determine the ability of bulk brain tissue data to capture microglial genes, we assessed the expression levels of our microglial signature genes in each of the 7 AMP‐AD bulk brain RNAseq datasets. Of the 1971 microglial signature genes in our study, 37–47% were captured in these bulk brain datasets (Figure S3A‐B). Our microglial signature genes comprised 3.6–4.5% of the expressed bulk brain transcriptome, consistent with prior estimations (Mathys et al., 2019; Wang et al., 2020). We next compared bulk microglia RNAseq transcript levels to that obtained from bulk tissue RNAseq of neurosurgical fresh brain tissue samples. Bulk fresh brain tissue does not capture all microglial marker genes, as demonstrated by the low correlation between bulk tissue and bulk microglia data (*R* = 0.46, *p *= 0.94; Figures S3C, S4, Table S6). This reiterates the need for complementary single‐cell type data to deconvolute cell type‐specific expression. We provide the list of microglial signature genes that are also expressed at high levels in bulk brain tissue data (Table S5), which can serve as a validated resource for microglial signature gene markers in bulk RNAseq datasets.

To determine how the microglial signature in this study compared to previously published signatures, we performed hypergeometric tests of overrepresentation with Galatro et al. (2017), Gosselin et al. (2017) and Olah et al. (2018) studies. Significant overlap was observed across all datasets, with 350 genes common to all datasets (Figure 1c‐d, Tables S3–S4, S8). This comprised several established microglial marker genes, including *P2RY12*, *TMEM119*, and *CX3CR1*. The most significant overlap was shared with Gosselin, et al. (Gosselin et al., 2017) signature [OR = 19.6 (17.0‐Inf) *p* = 3.8E‐261], where 49.7% of their genes were also present in our signature, and 22% of ours in their signature. Gosselin et al. (2017) samples were also obtained from neurosurgical tissue resections like our cohort and are unlike Galatro et al. (2017) and Olah et al. (2018) samples that were harvested during autopsy. Although there appears to be a common set of microglial genes consistent across signatures, each also harbors many unique genes, which could be due to study or individual specific differences.

### 2.2 Transcriptional profiling of microglia discovers co‐expression networks and implicates lipid and carbohydrate metabolism pathways associated with age, sex, and *APOE*

**FIGURE 2:**
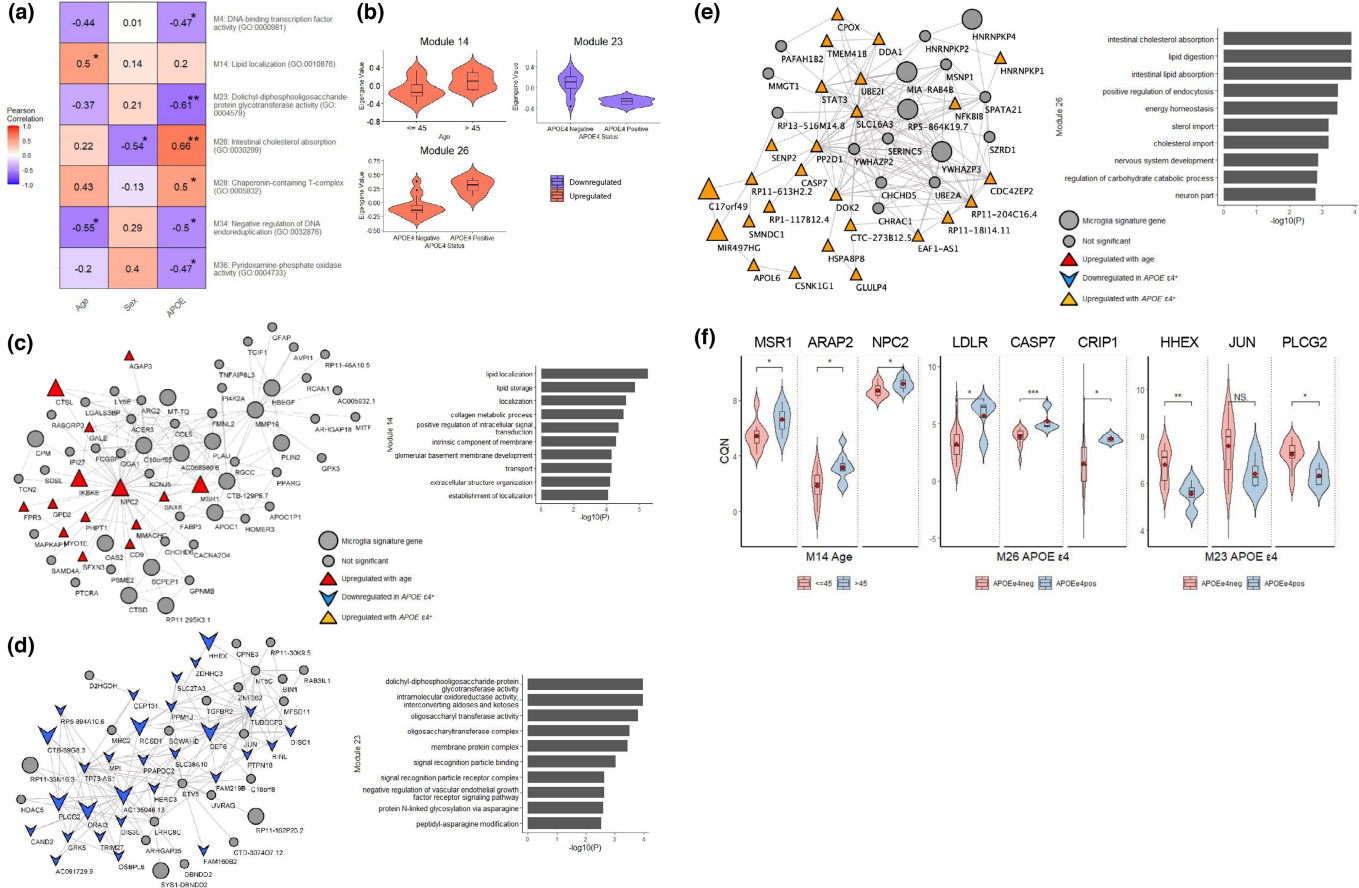
Age, sex and *APOE* ε4 pathway correlations in bulk microglia. (a) Heatmap showing correlation of age, sex and *APOE* ε4 status with WGCNA module eigengenes (MEs) significantly associated (*p *< 0.05) with traits, with top GO terms listed for each module. (b) Module eigengenes stratified by age or *APOE* ε4 status. (c) Module M14 gene co‐expression network, with genes of interest highlighted according to the key. Genes upregulated with age shown in red triangle (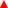). Bar plot of top 10 significant GO terms (*p *< 0.05) for this module. (d) Module 23 gene co‐expression network, with genes downregulated in *APOE* ε4 carriers shown in blue arrow (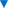). Bar plot of top 10 significant GO terms (*p *< 0.05) for this module. (e) Module 26 gene co‐expression network, with genes upregulated in *APOE* ε4 carriers shown in orange triangle (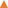). Bar plot of top 10 significant GO terms (*p *< 0.05) for this module. (f) Violin plots showing expression of key genes in modules, stratified by age or *APOE*. *p < 0.05; **p < 0.01; ***p < 0.001

We generated gene co‐expression networks using WGCNA (Langfelder & Horvath, 2008) to reduce number of tests and increase power to detect genetic associations with age, sex, and *APOE*. We identified 7 modules with significant associations (Figure 2; Figure S5; Table S9). Modules ME14 and ME34 associated with age, however, in opposite directions. ME14 was enriched for genes involved in the lipid localization pathway that were upregulated with age (*R* = 0.50, *p *= 0.03; Figure 2a‐c). ME34, enriched for DNA endoreduplication genes, had negative association with both age (*R* = −0.55, *p *= 0.01) and *APOE*‐ε4 (*R* = −0.50, *p *= 0.03), indicating that microglial transcripts involved in this pathway are downregulated with aging and in *APOE*‐ε4 carriers (Figure 2a). Several other modules also associated with *APOE*‐ε4, in either direction. The only module associated with sex was ME26, which was downregulated in females (*R* = −0.54, *p *= 0.02), and enriched for genes involved in cholesterol absorption and lipid digestion. This module also had the most significant association with *APOE*, in the positive direction with presence of *APOE*‐ε4 (*R* = 0.66, *p *= 0.002; Figure 2a,b,e). Of the *APOE*‐associated modules, ME23 had the second most significant association (*R* = −0.61, *p *= 0.006) and was enriched for carbohydrate metabolism genes (Figure 2a,b,d). Given recent discoveries in microglial immunometabolism (Bernier et al., 2020; Chausse et al., 2020; Loving & Bruce, 2020; Marschallinger et al., 2020), we focused on ME14, ME23, and ME26 that are enriched for lipid and carbohydrate metabolism genes.

ME14 co‐expression network (Figure 2c) hub genes *NPC2*, *MSR1*, and *PLAU* are also microglial signature genes in our study and known to be involved in microglial functions (Butovsky & Weiner, 2018; Colombo et al., 2021; Cunningham et al., 2009; DePaula‐Silva et al., 2019; El Khoury et al., 1998]; Mehra et al., 2016). Several disease‐associated microglial (DAM) markers are also present in this network, including *CD9*, *ARAP2*, and *MYO1E* (Keren‐Shaul et al., 2017; Rangaraju et al., 2018a; Sobue et al., 2021) that are increased with aging, implicating activated microglial lipid localization pathways in aging (Figure 2f). Several genes in this module were also previously linked to neurodegeneration, including *MYO1E* (Gerrits et al., 2021]; Rangaraju et al., 2018b), *CTSL*, (Cermak et al., 2016) and *UNC5B* (Ahn et al., 2020; Xu et al., 2016). Due to the nature of the tissue, we compared expression levels of the key module genes within tumor and epilepsy samples (Figure S6). We observed significant differences due to diagnosis in *NPC2*, *PLAU*, *APOC1*, and *IKBKE*; therefore, it is important to note that some of their associations may be confounded due to the disease state.

Our microglial signature (Tables S1–S3) had significant overrepresentation of the age‐associated ME14 genes (Table S9; OR = 1.55 [95% CI = 1.23‐INF], *p *= 0.001), highlighting age‐related increases in microglial signature genes. Galatro et al. (2017) and Olah et al. (2018) also reported age‐related microglial signatures. Comparison of ME14 genes revealed significant overlap with Olah et al. (2018) (OR = 1.34 [95% CI = 1.05‐INF] *p *= 0.03), but not with Galatro et al. (2017) microglial aging signature genes (OR = 1.09 [95% CI = 0.81‐INF] *p *= 0.33).

ME26 cholesterol metabolism pathway genes exhibited reduced expression in males and were elevated in *APOE*‐ε4 carriers (Figure 2a,b). This module contains known microglial genes *LDLR*, *CD36*, and *CRIP1* (Figure 2e,f). Assessment of individual ME26 network genes revealed *C17orf49*, *RP11*‐*589P10*.*7*, and *MIR497HG* to be the only microglial signature genes in this network to be associated with both sex and *APOE* (Figure 2e). Other microglial signature genes in ME26 associated with only sex or only *APOE*, suggesting that these traits may have independent effects on expression of some microglial genes. Several *APOE*‐associated genes in ME26 were previously implicated in AD, including *CASP7* (Ayers et al., 2016; Zhang et al., 2019) and *LDLR* (Katsouri & Georgopoulos, 2011; Lämsä et al., 2008; Figure 2f).

Carbohydrate metabolism gene enriched module ME23 is downregulated in *APOE*‐ε4 carriers (Figure 2a,b,d). AD risk genes *BIN1* (Crotti et al., 2019) and *PLCG2* (Sims et al., 2017) are present in this network, which have both been implicated in microglial dysfunction in neurodegeneration (Figure 2d).

### 2.3 Single‐cell transcriptome reveals specific subtypes of microglia

**FIGURE 3:**
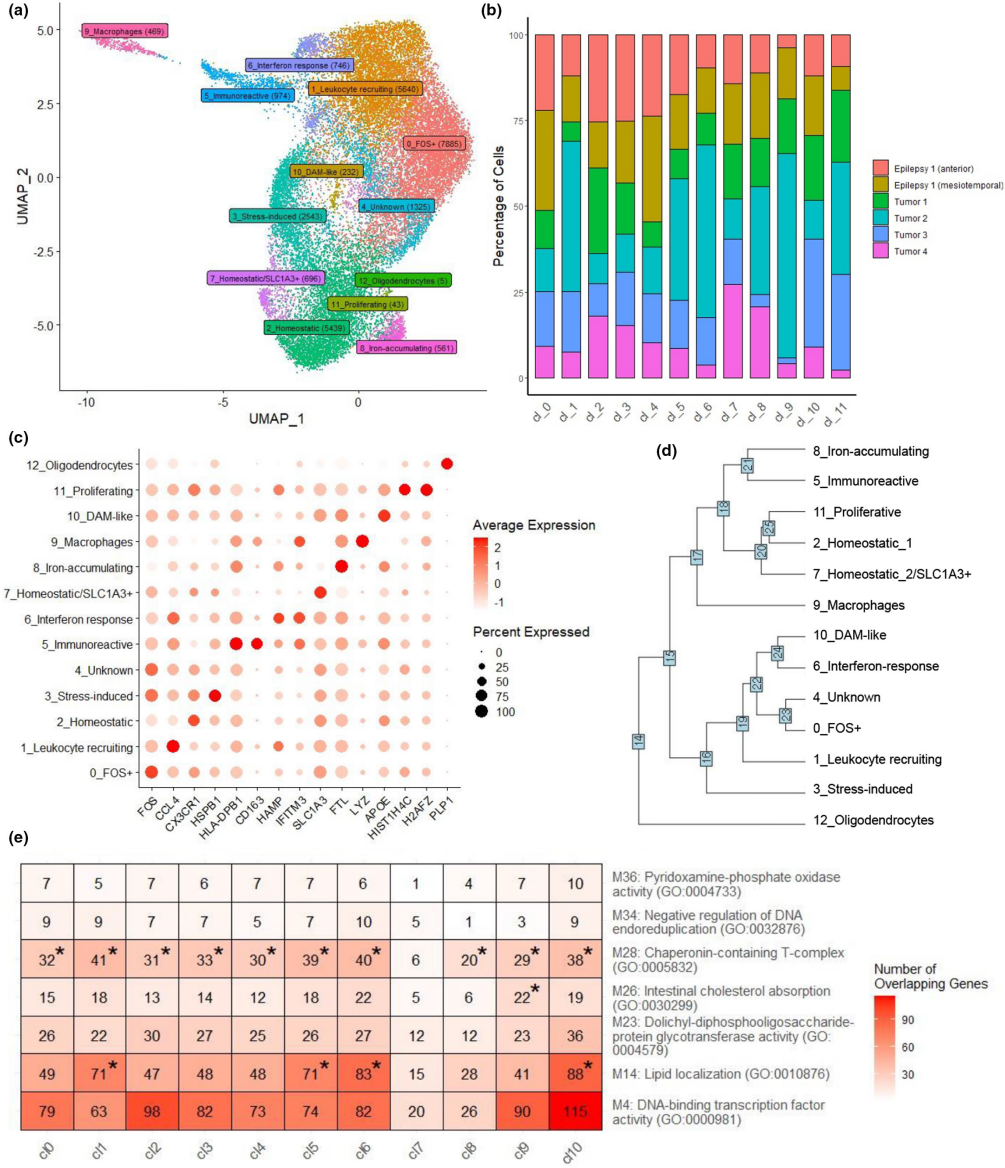
Single‐cell microglial data. (a) UMAP of clustered cells annotated with putative subtypes using cell type markers from the literature. (b) Stacked bar plot showing the distribution of cells across the clusters. (c) Dot plot showing the expression of key significant module genes across clusters. (d) Hierarchical clustering to highlight relationships between clusters. (e) Hypergeometric distribution of enrichment between module genes and clusters, showing number of overlapping genes. * Represents module genes that were significantly enriched in the cluster (*p* < 0.05)

To uncover distinct microglial subtypes, a subset of sorted microglial samples from neurosurgical brain tissue underwent single‐cell expression profiling. We obtained 26,558 cells from 5 unique individuals, including one individual who underwent epilepsy surgery and had samples from two brain regions (Table 1). Analysis of the scRNAseq data from these samples revealed 13 distinct cell clusters, which were annotated using established neuronal and glial marker genes from the literature (Darmanis et al., 2015; Keren‐Shaul et al., 2017; Masuda et al., 2019; Mathys et al., 2019; Olah et al., 2020; Rangaraju et al., 2018a; Sankowski et al., 2019; Zhou et al., 2020; Figure 3a, Tables S10‐S16). Myeloid markers (*AIF1*, *PTPRC*, and *C1QA*) were detected in all clusters except cluster 12 which expressed oligodendrocyte markers (*PLP1*, *MBP*, and *MOBP*). Cluster 9 expressed macrophage‐specific markers (*VCAN*, *FCN1*, *CRIP1*, and *S100A8*). These two clusters comprised <3% of all cells, indicating that our sorted samples represent a very pure microglial population. Each myeloid cluster had cellular contributions from all samples, albeit with some variability in their proportions, likely due to intrinsic differences between individuals (Figure 3b, Table S11). For these samples, the most marked difference was observed for macrophages (cluster 9) and homeostatic microglia (cluster 2), which had greater contributions from the mesiotemporal and anterior temporal regions, respectively. This could be due to the proximity of the mesiotemporal sample to the disease‐affected region.

We characterized the microglial clusters by their expression of established microglial subtype markers (Figure 3c, Figure S7) and their most significant marker genes (Figure S8). Homeostatic (*TMEM119*, *P2RY12*, and *CX3CR1*; Masuda et al., 2019; Rangaraju et al., 2018a; Sankowski et al., 2019; Zhou et al., 2020), pro‐inflammatory (*CCL2*, *CCL4*, Masuda et al., 2019; Sankowski et al., 2019) and DAM markers (*APOE*, *C1QA*, and *C1QB*; Hammond et al., 2019; Keren‐Shaul et al., 2017; Olah et al., 2020; Sankowski et al., 2019) were observed in clusters 2, 1/6, and 10, respectively. Cluster marker genes are defined as those expressed in at least 70% of the cells in the cluster with log fold change >0.6 and *q* < 0.05 in comparison to all other clusters. Expression levels of the top marker genes per cluster are shown (Figure 3c; Figure S8; Table S12). Most of these markers are distinct to a single cluster, although some clusters appeared to have similarities in their marker expressions. To define the proximity of their transcriptional profiles, we performed hierarchical clustering of the microglial clusters (Figure 3d). We determined that the homeostatic microglia cluster 2 was transcriptionally closest to clusters 7 and 11, which may represent subtypes of homeostatic microglia. Cluster 11 is enriched for markers of cell proliferation (*STMN1*, *H2AFZ*, *PCNA*, and *MKI67*), some of which were also observed by Olah et al. (2020), suggesting that these could be proliferating microglia. Clusters 1 and 6 both expressed inflammatory chemokines *CCL2* and *CCL4* and anti‐inflammatory molecule *EGR2*; however, cluster 6 was more closely related to DAM, whereas cluster 1 (named as Leukocyte‐recruiting cluster) represented a more pro‐inflammatory signature with greater expression of *IL6* and *TNFα*. Cluster 6 (named as interferon‐response cluster) also highly expressed interferon‐related marker *IFITM3* and *ISG15*, also observed in a cluster by Olah et al. (2020), which they defined as an interferon response‐enriched subset. The upregulation of chemokines and interleukins in these clusters suggest that they could be involved in recruitment of other immune cells. These findings highlight different transcriptional profiles for these two inflammatory clusters that may represent distinct activated microglia subtypes. Cluster 3 highly expressed heat shock protein *HSPA1A*, an immediate early gene (Schmunk et al., 2020) reportedly involved in antigen processing (Aung et al., 2012), response to stress and injury and exhibiting decreased gene expression in multiple sclerosis patients (Gandhi et al., 2010; Satoh et al., 2005). Several were upregulated in this cluster, suggesting that this may represent cells that underwent dissociation‐induced stress (Sankowski et al., 2019). Several of the clusters did not express well known existing cell type markers. Clusters 5/8 and FOS^+^0/4 were transcriptionally closest to one another (Figure 3d). Cluster 5 has distinct expression of immunoreactive marker *CD163*, which was not observed in other subsets except macrophages. Several *HLA* genes are also highly expressed in this cluster, suggesting that these may be immunoreactive microglia (Hendrickx et al., 2017). Cluster 8 marker FTL has recently been used to characterize iron‐accumulating microglia (Kenkhuis et al., 2021). Our findings highlight transcriptional profiles for known microglial clusters, describe the transcriptional proximity of these clusters and suggest that less well‐defined clusters could potentially represent novel or intermediate transcriptional states of microglia. Our microglial signature was significantly enriched in more clusters expressing more activated markers (Table S13), implicating this as the dominant expression profile within our samples. However, there is also enrichment of homeostatic cluster 2, demonstrating that we have not only captured activated cells as might be expected due to the nature of the tissue.

To determine whether the bulk microglial co‐expression networks (Figure 2a,c‐e, Figure S5) were representative of microglial subtypes, we performed enrichment analyses of the module genes within the myeloid clusters with sufficient cell numbers (Figure 3e; full enrichment statistics provided in Table S15). Age‐associated co‐expression network ME14, implicated in lipid metabolism, was significantly enriched in interferon‐response (cluster 6) and DAM (cluster 10) clusters. Genes within module 28, which was significantly upregulated with *APOE*‐ε4, had statistically significant enrichment in all clusters except cluster 7. There was no statistically significant enrichment for any of the other microglial modules that had significant age, sex, or *APOE* associations, suggesting that these factors may have ubiquitous effects on most microglial subtypes. Some of the remaining microglial co‐expression networks had distinct patterns of cluster enrichment (Figure S9), suggesting that some but not all networks could be representative of distinct microglial subtypes.

### 2.4 Meta‐analyses with published datasets support age, sex, and *APOE* associations

**FIGURE 4:**
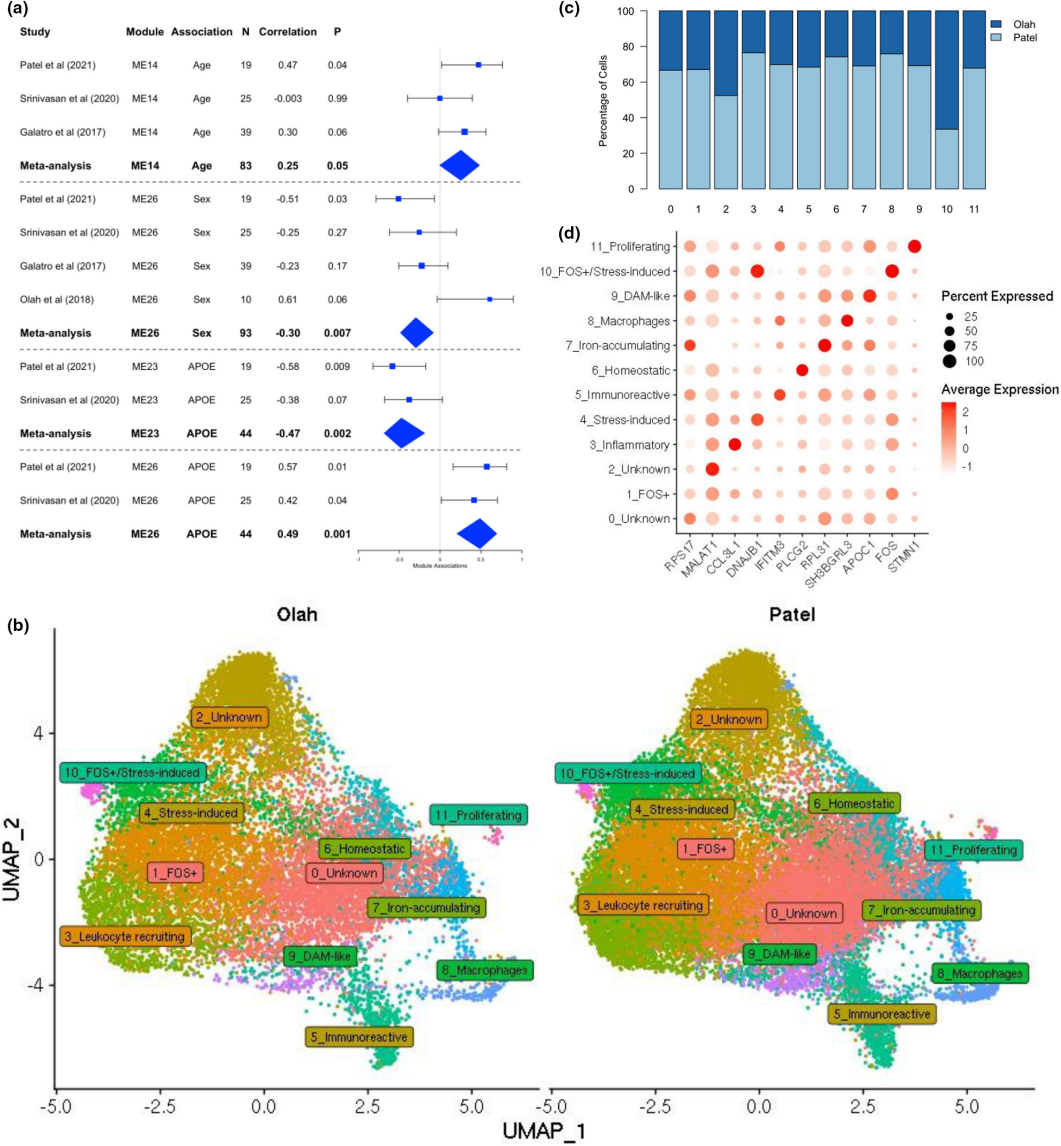
Meta‐analysis with published datasets. (a) Forest plots of module eigengene correlations across datasets and meta‐analyzed. (b) Integrated UMAP of our and Olah et al. (2020) single‐cell data, split by dataset. (c) Stacked bar plot showing the distribution of cells across the clusters. (d) Dot plot showing the expression of key significant module genes across clusters

Each dataset was individually processed through the same MAPR‐seq pipeline for quality control to minimize variability due to data processing. Meta‐analysis of WGCNA results was performed by coercing the external dataset co‐expression networks onto our existing co‐expression networks. The forest plot in Figure 4a highlights the individual and combined associations for networks of interest across the datasets where age, sex, or *APOE* information was available (Table S17). For module ME14 associations with age, Galatro et al. (2017) also exhibited a similar direction of effect (*R* = 0.30, *p *= 0.06), whereas Srinivasan et al. (2020) did not show any association (*R* = −0.003, *p *= 0.99), likely due to a higher median age of individuals in the study. When meta‐analyzed, sex‐associated ME26 genes were significantly downregulated in males in all datasets, supporting our finding. This module was also significantly associated with *APOE* e4 carriers in the Srinivasan dataset (*R* = 0.42, *p *= 0.04). However, Srinivasan samples were inversely correlated with *APOE* for ME23 in comparison to our data (*R* = 0.61, *p *= 0.07). Srinivasan et al. (2020) was inversely correlated with age and *APOE* for ME23, but not with sex or *APOE* for ME26. Overall, it appears that the direction of effects was similar for correlation with these traits in most datasets (Figure S10).

We subsequently integrated microglial single‐cell data from Olah et al. (2020) (*n* = 9, 15,819 cells) with our samples (*n* = 6, 26,856 cells) to show how these single‐cell datasets compared. The UMAP in Figure 4b is split by study and shows relatively even contributions to each cluster from both datasets, also observed in the stacked bar plot in Figure 4c. However, a greater number of Olah cells were observed in cluster 10, which had similar expression patterns to our original FOS^+^ cluster 0. Hypergeometric tests of enrichment showed significant overlap between several of the original and integrated clusters, showcasing the high congruence between our data and Olah et al (Tables S18–S20). To further identify where our individual cells clustered when combined with the Olah dataset, we overlaid the cell IDs from the original clusters to the integrated dataset and calculated the percentage overlap (Table S20). A larger proportion of cells from Olah et al expressed markers of activation than homeostatic genes, indicating a greater enrichment for activated microglia. Many clusters retained their identity, as evidenced by the dot plot (Figure 4d) which shows some of the same top marker genes to that in Figure 3c. Additionally, enrichment of our microglial signature in the integrated single‐cell dataset was observed in several clusters, many of which highly expressed markers of activated microglia (Table S18). This is similar to what we observed in our single‐cell data (Table S13), supporting the notion that our microglial signature is enriched for activated cells.

## 3 DISCUSSION

Given their critical functions in maintaining homeostasis in the central nervous system (CNS) in health and their multifaceted roles during neurological diseases (Hickman et al., 2018; Li & Barres, 2018), understanding the biology of microglia and characterizing microglial subtypes is essential. Large‐scale studies in bulk brain tissue (Allen et al., 2016]; De Jager et al., 2018; Wang et al., 2018) have been instrumental in establishing transcriptional profiles in health and neurodegenerative diseases. Although these studies yielded information on brain expression signatures and uncovered perturbed pathways and molecules implicated in Alzheimer's disease and other neurological disorders (Allen et al., 2018a, 2018b; Mostafavi et al., 2018; Neff et al., 2021), they are limited in their ability to provide cell type‐specific transcriptional outcomes, especially for less abundant CNS cells such as microglia (Wang et al., 2020). Analytic deconvolution approaches began to leverage these bulk tissue transcriptome datasets to estimate cell type‐specific expression profiles (McKenzie et al., 2018; Wang et al., 2020), but the accuracy of these methods relies on the availability of high‐quality single cell‐type datasets. Such microglia‐specific transcriptome datasets are gradually emerging (Galatro et al., 2017; Gosselin et al., 2017; Olah et al., 2018, 2020), although the numbers of unique samples assessed remain limited given the arduous nature of collecting fresh human brain tissue. Additionally, comparative assessment of bulk brain vs. single cell‐type bulk microglia vs. single‐cell microglia studies are still rare (Alsema et al., 2020; Olah et al., 2020; Srinivasan et al., 2020). To our knowledge, there are no studies that evaluate human microglial transcriptome using all three approaches, as in our study. Further, investigations on effects of genetic and other factors on microglial transcriptional signatures in humans is likewise sparse, with the exception of age‐related effects assessed in a few studies (Galatro et al., 2017; Gosselin et al., 2017; Olah et al., 2018). Finally, unlike in bulk tissue studies (Allen et al., 2018a, 2018b; McKenzie et al., 2018; Mostafavi et al., 2018; Neff et al., 2021), microglia‐specific co‐expression networks, their molecular signatures and functional implications have not been evaluated.

In this study, we sought to overcome these knowledge gaps by characterizing the transcriptome of sorted bulk and single‐cell microglial populations isolated from fresh human brain tissue. We identified a robust microglial signature comprising 1971 genes enriched for immune‐related functions. These signature genes were selected due to their consistently higher expression levels in our sorted bulk microglial transcriptome in comparison with 7 different bulk brain tissue datasets from 6 different regions (Allen et al., 2016; De Jager et al., 2018; Wang et al., 2018). We also compared sorted bulk microglia to bulk fresh brain tissue and identified transcripts that are expressed in both. The microglial signature genes that are also reliably detected in bulk brain tissue represent a validated list of microglial markers that can be utilized in bulk brain tissue transcriptome analytic deconvolution studies (McKenzie et al., 2018; Wang et al., 2020).

Our microglial signature significantly overlapped with other signatures from bulk microglia previously reported by Galatro et al. (2017), Gosselin et al. (2017) and Olah et al. (2018), implicating a core set of genes consistently expressed in this cell type. However, there were additional genes unique to each signature, likely to be driven by factors such as patient demographics or study differences. Galatro et al. (2017) and Olah et al. (2018) both also reported age‐related microglial expression signatures. We found significant overlap of our age‐associated microglial gene expression module ME14 genes with the latter, which was also enriched for our microglial signature. This indicates that bulk microglial profiles can effectively capture genes affected by aging in microglia.

We leveraged the co‐expression network structure of sorted bulk microglia to further explore whether microglial subsets were associated with age, sex, or *APOE*‐ε4. To our knowledge, sex differences in microglial transcriptome were previously studied only in mice (Frigerio et al., 2019; Nelson et al., 2017; Stephen et al., 2019); however, *APOE* genotype‐specific microglial interactions with amyloid plaques have been previously observed in mice (Shi et al., 2019; Stephen et al., 2019) and humans (Nguyen et al., 2020). We identified two network modules associated with age, one with sex and six with *APOE*‐ε4. We observed that two modules, ME14 that is positively associated with increased age; and ME26 that is positively associated with both *APOE*‐ε4 and female sex, were both enriched for lipid metabolism biological terms (Chausse et al., 2020; Loving & Bruce, 2020; Marschallinger et al., 2020). Module ME14 included genes involved in lipid localization and storage pathways (*PLIN2*, *IL6*, *LPL*, *MSR1*, *ENPP1*, *PPARG*, *PTPN2*, *SOAT1*, *IKBKE*) and ME26 had lipid digestion/cholesterol transport pathway genes (*CD36*, *LDLR*). Both modules harbored known microglial genes (*LDLR*, *CD36*, *CRIP1*, *NPC2*, *MSR1*, *PLAU)* and those that are included in our microglial signature (*PLIN2*, *IL6*, *MSR1*, *SOAT1*, *IKBKE*, *NPC2*, *PLAU*).

Comparing the sorted bulk microglial network modules to scRNAseq microglial clusters, we determined that ME14 genes were significantly over‐represented in interferon‐response cluster 6 and disease‐associated microglia (DAM) cluster 10. In our study, DAM cluster 10 included *APOE*, *APOC1*, *ASAH1*, and *CTSD*. Of these *APOE* (Leduc et al., 2010; Loving & Bruce, 2020; Yamazaki et al., 2019), *APOC1* and *ASAH1* (Paciotti et al., 2020) are involved in lipid metabolism and neurodegenerative diseases. *APOE* (Hammond et al., 2019; Keren‐Shaul et al., 2017; Krasemann et al., 2017), *APOC1*(Hammond et al., 2019 *)*, and *CTSD* (Keren‐Shaul et al., 2017) were also signature genes in mouse models of neurodegenerative diseases (Keren‐Shaul et al., 2017; Krasemann et al., 2017) or aging (Hammond et al., 2019). Our interferon‐response cluster 6 also included genes associated with mice microglial neurodegenerative (*FTH1* Keren‐Shaul et al. (2017)) or aging signatures (*CCL4* Hammond et al. (2019)), as well as *IFITM3* (Marschallinger et al., 2020) and *GOLGA4* (Marschallinger et al., 2020), previously shown to be upregulated in aging lipid droplet accumulating microglia (Marschallinger et al., 2020). Our findings that integrate human sorted bulk RNAseq and scRNAseq data, support a model where aging human microglia transition to a pro‐inflammatory and disease‐associated transcriptional profile, which is also associated with perturbations in lipid metabolism in these cells.

There is increasing evidence that tightly controlled lipid metabolism is essential to the functions of microglia during development and homeostatic functions of adulthood and may be disrupted in aging and disease (Chausse et al., 2020; Loving & Bruce, 2020). The complex interactions between microglial lipid metabolism and its cellular functions rely on lipid sensing by microglial receptors such as CD36 and TREM2 and uptake of lipids, including LDL and APOE (Chausse et al., 2020; Loving & Bruce, 2020). These interactions are necessary for microglia to become activated and perform functions including phagocytosis of myelin (Nugent et al., 2020) and misfolded proteins like amyloid ß (Yeh et al., 2016), cytokine release, migration and proliferation (Bernier et al., 2020; Chausse et al., 2020). Studies primarily focused on *in vitro* and animal models suggest disruption of the microglial immunometabolism and assumption of a pro‐inflammatory phenotype with aging (Hammond et al., 2019; Koellhoffer et al., 2017; Marschallinger et al., 2020; Norden & Godbout, 2013) and diseases including multiple sclerosis (MS) and Alzheimer's disease (Keren‐Shaul et al., 2017; Krasemann et al., 2017; Ulland et al., 2017). Interestingly, microglial lipid droplet accumulation has been demonstrated under all these conditions (Chausse et al., 2020; Loving & Bruce, 2020; Marschallinger et al., 2020; Nugent et al., 2020) and lipid droplet accumulating microglia in aging mice were shown to have a unique transcriptional state (Marschallinger et al., 2020). Our findings in sorted cells from fresh human brain tissue provide transcriptional evidence for immunometabolism changes and pro‐inflammatory phenotype with microglial aging, thereby contributing essential complementary data from humans for this cell type.

Besides module ME14, we determined that ME26 is also enriched for lipid metabolism genes. ME26 module expression is higher in both *APOE*‐ε4 and female sex; however, we note that in our sorted bulk microglia RNAseq samples, there were no male *APOE*‐ε4 carriers. Therefore, the distinct influence of sex and *APOE* on the expression of this module remains to be established. *APOE*‐ε4, a major risk factor for Alzheimer's disease, has the lowest lipid binding efficiency compared with other *APOE* isoforms (Chausse et al., 2020). Increased cholesterol accumulation has been reported in both iPSC‐driven astrocytes from *APOE*‐ε4 carriers (Lin et al., 2018) and in Apoe‐deficient microglia (Nugent et al., 2020). These findings collectively support a role for *APOE*‐ε4 associated microglial transcriptional changes and disrupted cholesterol metabolism. Using our sorted microglia RNAseq data, we identified five additional modules that associate with *APOE*‐ε4, one in a positive direction (ME28) and four negatively (ME4, ME23, ME34, and ME36). Of these, module ME23 had the second most significant *APOE*‐ε4 association after ME26. Interestingly, ME23 was enriched for carbohydrate metabolism biological processes, which are also tightly regulated in microglia (Bernier et al., 2020). Module ME23 harbors known AD risk genes *BIN1* and *PLCG2*, where the latter is a microglial gene that modulates signaling through *TREM2* (Andreone et al., 2020) and also a hub gene in this module. ME23 genes *BIN1*, *JUN*, and *TGFBR2* were found to be reduced in a mouse microglial neurodegenerative phenotype gene signature (Krasemann et al., 2017). These findings further demonstrate the consistency of our human microglial data with that from mouse models and supports perturbed microglial immunometabolism as a potential pathogenic mechanism in neurodegeneration. Many of the meta‐analysis results comprising published datasets from Galatro et al. (2017), Olah et al. (2018), and Srinivasan et al. (2020) supported the directions of effects we had observed in our study despite small sample numbers.

In addition to analyzing gene expression modules from sorted bulk microglia, we also identified microglial clusters from sorted microglial scRNAseq data. To our knowledge, there are only two prior publications of scRNAseq characterizations on human microglia (Masuda et al., 2019; Olah et al., 2020). Masuda et al. (2019) analyzed 1,602 microglia isolated from 5 control and 5 MS patient brains, compared their findings to those from mice demonstrating clusters that are common and others that are species‐specific. Olah et al. assessed 16,242 microglia from 17 individuals and characterized subclusters of microglia from patients with mild cognitive impairment, AD, and epilepsy (Olah et al., 2020). Our scRNAseq dataset is from 5 unique individuals comprising 26,558 cells, 99.98% of which have myeloid markers. A small cluster highly enriched for mature oligodendrocyte and some neuronal marker genes were observed (Table S11). As there were only 5 cells in this cluster, this is likely to denote contamination from the sorting procedures. We identified microglial clusters that share characteristics of those previously reported in mice (Keren‐Shaul et al., 2017) and humans (Nguyen et al., 2020; Olah et al., 2020), such as DAM. We also uncovered clusters that had not been previously characterized in the literature, including cluster 7, exhibiting high expression levels of astrocytic marker gene *SLC1A3*. Microglial expression of *SLC1A3* was previously shown to occur in mice and humans especially in disease states (Chrétien et al., 2004; Grassivaro et al., 2020; Wilhelmsson et al., 2017). We also leveraged these scRNAseq data to further characterize the sorted bulk microglial expression modules. Integration of our samples with Olah et al. allowed us to explore microglial subtypes at single‐cell resolution with increased power. We found that even in this larger dataset, our microglial signature was similarly enriched in the more activated subtype clusters, demonstrating the robustness of this signature across datasets. The homogeneity in clustering highlighted shared expression patterns between cells from both datasets, providing further support for the putative microglial subtypes we identified here. Hence, our microglial scRNAseq data contribute further to the emerging single‐cell landscape of this cell type.

Activated microglia are a hallmark in both brain cancers and temporal lobe epilepsy in response to the inflammation (Darmanis et al., 2017; Kaminska et al., 2021; Kinoshita & Koyama, 2021; Morin‐Brureau et al., 2018; Ochocka et al., 2021). We acknowledge that the tissue used in this study is sourced from tumor or epileptogenic tissue adjacent regions in individuals with diagnoses of brain tumors or epilepsy, meaning it is not entirely healthy. Therefore, it is possible that the activated subtypes we identify here may be due to the nature of the tissue. Additionally, sex‐specific expression differences have been observed in glioma‐activated microglia (Ochocka et al., 2021). Nonetheless, we did not observe significant differences in most genes we highlighted here due to diagnosis (Figure S5). There is also a large cluster of homeostatic microglia present in these samples, indicating that not all cells are activated. While the focus of our work is AD, these findings also have potential relevance in the fields of cancers, epilepsy, and other diseases.

We recognize that our study has several limitations, primarily owing to the difficulty in obtaining high‐quality neurosurgical brain tissue, which leads to limited sample size and variability in tissue, diagnoses, and patient demographics. Even though we have utilized tissue surgically separated from disease tissue, the samples are from epilepsy and various brain tumor patients representing multiple diagnoses. We isolated microglia using an approach which should minimize activation; however, we cannot definitively rule out stress‐induced transcriptomic changes during isolation. Despite these caveats, we could identify microglial co‐expression modules and subclusters with multiple features that are consistent with prior publications from model systems (Hammond et al., 2019; Keren‐Shaul et al., 2017; Krasemann et al., 2017; Marschallinger et al., 2020). Our scRNAseq clusters have contributions from both tumor and epilepsy samples, suggesting that our findings are unlikely to be driven by any one diagnoses. Furthermore, there are few published studies using fresh brain tissue to study microglia and thus our integrated single‐cell data showing a homogenous cluster of microglia highlights the robustness of the methodology.

In summary, our study on sorted bulk microglia RNAseq and scRNAseq from fresh brain tissue yield several key findings. We identify a microglial gene signature from sorted bulk microglia, characterize its expression in bulk brain RNAseq across 7 datasets comprising 6 regions, in bulk fresh brain RNAseq and in microglial scRNAseq subtype clusters. This signature provides a well‐characterized resource which can be utilized in analytic deconvolution studies of bulk transcriptome data (McKenzie et al., 2018; Wang et al., 2020). We uncovered microglial gene expression modules associated with age, sex and/or *APOE*‐ε4. Modules with age and *APOE*‐ε4‐associated transcriptional changes implicate microglial lipid and carbohydrate metabolism perturbations and microglial activation. Microglial scRNAseq data highlight the transcriptional complexity of this cell type, reveal both known and novel cell types, and demonstrate utility of this data in characterizing sorted bulk RNAseq data. These findings provide support for the emerging microglial immunometabolism (Bernier et al., 2020; Chausse et al., 2020) pathway as a plausible therapeutic target in aging‐related disorders; and provide a well‐characterized human transcriptome resource for the research community on this cell type with central roles in health and disease (Masuda et al., 2020).

## 4 MATERIALS AND METHODS

### 4.1 Patient samples

Fresh human brain tissue was obtained from patients undergoing neurosurgical procedures for epilepsy or tumor resection. Tissues determined to be grossly unaffected by the primary disease process were utilized for the present study (Figure 1). Patient samples were transported from the operating room to the laboratory in 1X DPBS (Thermo Fisher; 14287080) for processing within 1–2 h of resection. Human tissue was collected with informed consent prior to surgery and all procedures were approved by the Mayo Clinic Institutional Review Board and are HIPAA compliant.

### 4.2 Tissue dissociation

Tissue was dissected to remove necrotic tissue, white matter and excess vascular tissue, to retain only cortical gray matter. The remaining tissue was cut into sagittal slices and weighed before being processed using the Adult Brain Dissociation Kit (Miltenyi; 130‐107‐677) as per the manufacturer's protocol. Debris removal (Miltenyi; 130‐109‐398) and red blood cell lysis (Miltenyi; 130‐094‐183) were also performed. All procedures were carried out on ice. The resulting homogenate was filtered through a 70‐µm filter before proceeding.

### 4.3 Magnetic‐activated cell sorting (MACS)

The cell suspension was first enriched for CD11b^+^ cells by incubating with anti‐CD11b microbeads (Miltenyi; 130–049–601 clone M1/70) for 15 minutes according to manufacturer's recommendation. This was then washed with PB buffer (0.5% BSA, 1X PBS Ca^2+^/Mg^2+^ free pH 7.4) and filtered through a 70µm cell strainer before being applied to a large separation column (Miltenyi; 130‐042‐401) in a QuadroMACS separator magnet (Miltenyi; 130‐090‐976). The CD11b^+^ fraction was collected and resuspended in sterile filtered FACS staining buffer (1X PBS Ca^2+^/Mg^2+^ free, 0.5% BSA, 2% FBS, 3mM EDTA) for antibody staining.

### 4.4 Fluorescence‐activated cell sorting (FACS)

MACS sorted CD11b^+^ cells subsequently underwent FACS sorting to further purify the microglial population. The cell suspension was incubated in Human TruStain FcX blocking solution (1:20, Biolegend; 422302) at room temperature for 10 min. Subsequently, cells were stained with anti‐CD11b PE/Cy7 (1:100, Biolegend; 101206, M1/70) and anti‐CD45 Alexa Fluor 647 (1:100, Biolegend; 304056, HI30) antibodies for 30 min on ice. Following two washes with FACS staining buffer, SYTOX Green viability dye (1:1000, Thermo Fisher; S7020) was added for an additional 20 minutes. Single‐cell suspensions were filtered through a 40µm cell strainer (Falcon; 352235) before sorting on a BD FACS Aria II (BD Biosciences). CD11b^+^/CD45^intermediate^/SYTOX green^−^ cells were sorted directly into FACS staining buffer. Independently CD11b and CD45 are not microglial specific markers; however, gating cells with a CD11b+/CD45^intermediate^ signature allowed us to differentiate microglia from peripheral myeloid cells, such as macrophages, which are expected to be CD45^high^. An example of our FACS gating strategy is provided in Figure S2A. Briefly, we used cell lines expressing the markers of interest as positive controls to independently set the gates for CD11b (RAW 264.5 mouse macrophage cells) and CD45 (Jurkat cells) expression. CD45 intermediate cells were determined by selecting for mid‐level fluorescence within this gate. This was used to sort cells for both bulk and single‐cell RNAseq data generation to reduce the likelihood of peripheral myeloid cell contamination in the samples.

### 4.5 RNA isolation and sequencing

RNA from sorted microglial cells was isolated using the miRNeasy Serum/Plasma Kit (QIAGEN; 217184) and quantified on the Agilent BioAnalyzer 2100. cDNA libraries were generated using SMARTSeq2 v4 and Nextera Low Input Library Prep Kit. Samples were multiplexed and sequenced on the Illumina HiSeq 4000.

RNA from frozen bulk tissue was isolated using Trizol and chloroform, followed by DNase and clean up using the Rneasy Kit (QIAGEN; 74106). Libraries were generated using the TruSeq Stranded mRNA Library Prep Kit. Samples were multiplexed and sequenced on the Illumina HiSeq 4000. Base‐calling of all sequence data was performed using Illumina's RTA v2.7.7.

### 4.6 10X Single cell 3’ v3 library preparation of sorted microglia

Viability of MACS plus FACS sorted cells was assessed by Trypan blue (Gibco; 15250061) exclusion and cell density was determined using a hemocytometer prior to adjustment to target 4000–5000 cells. Cells were loaded onto a 10X Chromium chip and run on the GemCode Single Cell Instrument (10X Genomics) to generate single‐cell gel beads‐in‐emulsion (GEMs). Single‐cell RNAseq libraries were prepared using the Chromium Single Cell 3’ Gel Bead and Library Kit v2 and v3 (10X Genomics; 120237) and the Chromium i7 Multiplex Kit (10X Genomics; 120262) according to the manufacturer's instructions. Quality of cDNA libraries was determined using a BioAnalyzer 2100 DNA High Sensitivity assay (Agilent; 5067–4626) prior to sequencing one per lane on an Illumina HiSeq 4000.

### 4.7 Validation with quantitative real‐time PCR

Total RNA was extracted from sorted cells using the miRNeasy Serum/Plasma Kit (QIAGEN; 217184). Concentration and quality were assessed using the Agilent BioAnalyzer RNA 6000 Pico Kit (Agilent; 5067‐1514). RNA was normalized to 0.5ng/µl for cDNA synthesis using the SuperScript IV VILO Master Mix (Thermo Fisher; 11756050). TaqMan PreAmp Master Mix (Thermo Fisher; 4391128) was used to pre‐amplify the cDNA, followed by TaqMan Universal PCR Master Mix (Thermo Fisher; 4304437) with the following gene expression probes: *MOG*, *AQP4*, *THY1*, *PTPRC*, *ITGAM*, *P2RY12*, *PECAM1*, *CD34*, *GAPDH* (Thermo Fisher; Hs01555268_m1, Hs00242342_m1, Hs00174816_m1, Hs04189704_m1, Hs00355885_m1, Hs00224470_m1, Hs01065279_m1, Hs02576480_m1, Hs99999905_m1). RT‐qPCR was performed on a QuantStudio 7 Flex Real‐Time PCR System (Thermo Fisher) using a relative standard curve to quantify gene expression.

### 4.8 Validation with immunocytochemistry

Cultured cells were fixed with 4% paraformaldehyde (PFA) overnight at 4°C and blocked with blocking solution (10% BSA, 5% normal goat serum and 0.1% Triton‐X). Fixed cells were stained with anti‐TMEM119 (1:100, Biolegend; 853302) extracellular primary antibody with Goat anti‐mouse IgG secondary antibody conjugated to Alexa‐488 (1:100, Abcam; ab150113). Nuclei were stained with 1µg/ml DAPI (1:1000, Thermo Fisher; 62248) before mounting with AquaPoly Mount (Poly Sciences, 18606‐20). Images were acquired with a Zeiss LSM880 Confocal microscope using a Plan‐Apochromat 20x magnification and 0.8 objective at 1024 by 1024 pixels with a 0.5 microsecond pixel dwell time.

### 4.9 Data analysis

#### 4.9.1 Bulk microglia RNAseq processing

The MAPR‐Seq pipeline (Kalari et al., 2014) was used to align reads to human reference genome hg38 using STAR (Dobin et al., 2013) and count reads using featureCounts (Liao et al., 2014). FastQC was used for quality control (QC) of raw sequence reads, and RseQC was used for QC of mapped reads. Quality measures were examined including base calling quality, GC content, mapping statistics and sex check to ensure consistency between the recorded and inferred sex from expression of chromosome Y genes. Raw read counts were normalized using Conditional Quantile Normalization (CQN) to generate log_2_ scaled expression values via the Bioconductor package cqn, accounting for sequencing depth, gene length and GC content. Normalized CQN expression values were assessed using Principal components analysis (PCA) to identify and remove outliers, defined as greater than 4 standard deviations from the mean of the first two principal components. In addition, RPKM (reads per kilo bases per million) values were calculated.

### 4.10 Identification of a core microglial signature from bulk microglia data

To define a core microglial signature, we compared our bulk microglia data to cognitively normal control samples from the AMP‐AD bulk tissue transcriptome data from 7 different datasets representing 6 brain regions (Synapse ID: syn2580853); Mayo Clinic (Allen et al., 2016]) (cerebellum and superior temporal gyrus), Mount Sinai Brain Bank (Wang et al., 2018) BM10 (frontal pole), BM22 (superior temporal gyrus), BM36 (parahippocampal gyrus), BM44 (inferior frontal gyrus), and Rush University Religious Order Study‐Memory and Aging Project (ROSMAP; De Jager et al., 2018) (dorsolateral prefrontal cortex). Raw gene counts and metadata (see Acknowledgments) were obtained from the AMP‐AD RNAseq Harmonization study which had performed alignment and processing of all datasets and brain regions through a consensus pipeline (Wan et al., 2020). Samples were removed that had inconsistent sex between that indicated in metadata and that inferred from RNAseq expression; a RIN <5; were identified as gene expression outliers based on principal component analysis (PCA) (>4 standard deviation (SD) from mean PC1 or PC2), or missing metadata. In addition, duplicates (lowest read count sample removed) and those with rRNA (>5%) were removed from the MSBB datasets. Furthermore, samples not meeting neuropathological criteria as Alzheimer's disease (AD; McKhann et al., 1984) or control were excluded. To generate the microglial expression signature, only control samples from the AMP‐AD datasets were included. Raw read counts were normalized using Conditional Quantile Normalization (CQN). Log_2_ fold change and q‐values between each bulk tissue brain region and the bulk microglia profiles were calculated for each gene via linear regression using log_2_ (RPKM) without correction for covariates. Genes were filtered using a cutoff of 4‐fold greater expression in bulk microglia compared to each bulk tissue region and *q* < 0.05. Genes that passed these criteria and were significant in comparisons with all 7 bulk brain datasets determined the microglial signature. These signature genes were assessed for GO term enrichment with biological pathways using MsigDB. REViGO (Supek et al., 2011) tree plots were generated in R using GO terms obtained from MsigDB.

### 4.11 Weighted gene co‐expression network analysis

The CQN normalized expression values from bulk microglia were input to R WGCNA (Langfelder & Horvath, 2008) package v1.69. This analysis included 14,149 expressed genes, that is, median (CQN) > 2. Modules were identified, their eigengenes were calculated and merged if correlation of eigengenes > 0.7. Genes in the 40 modules identified were tested for GO term enrichment via WGCNA. Module membership (MM) for each gene was calculated as the correlation between expression of each gene and its module eigengene. Genes with MM ≥ 0.7 are considered the hub genes for the network. Gene co‐expression network plots were generated in Cytoscape v3.8 (http://www.cytoscape.org/). Each module eigengene was tested for association with age, sex and *APOE* e4 carrier status independently using Pearson correlation. Co‐expression network genes were annotated if they were significantly associated (*p* < 0.05) with the tested trait.

### 4.12 Over‐representation and correlation analyses

Hypergeometric testing was performed in R to determine the enrichment of a select set of genes in previously reported signatures, bulk tissue expressed genes, WGCNA modules or 10X single‐cell clusters. Correlation between bulk tissue and bulk microglial normalized CQN data was calculated using Spearman's rank correlation. Concordant and discordantly correlated genes were determined using the upper and lower quartiles from each dataset.

### 4.13 Single‐cell data analysis

For single‐cell RNA samples, 10X Genomics Cell Ranger Single Cell Software Suite v3.1.0 (Zheng et al., 2017) was used to demultiplex raw base call files generated from the sequencer into FASTQ files. Raw reads were aligned to human genome build GRCh38. Reads aligned to gene transcript locus were counted to generate raw UMI counts per gene per barcode for each sample. The raw UMI matrices were filtered to only keep barcodes with >500 UMIs and those that were classified as cells by Cell Ranger's cell calling algorithm.

Quality control, normalization, clustering, and marker gene identification were performed with Seurat v3 (Stuart et al., 2019), followed by annotation of clusters using established cell type markers. We kept (1) barcodes with >10% of UMI mapped to mitochondrial genome; (2) barcodes with <400 or >8000 detected genes; (3) barcodes with <500 or >46,425 mapped UMIs; (4) genes that are detected in <5 cells. These thresholds were determined by UMI or gene distribution to identify undetectable genes and outlier barcodes that may encode background, damaged or multiple cells. UMI counts of remaining cells and genes were normalized using NormalizeData function, which gave natural log transformed expression adjusted for total UMI counts in each cell. The top 2000 genes whose normalized expression varied the most across cells were identified through FindVariableFeatures function with default parameters. Using those genes, cells from 6 samples were integrated using functions FindIntegrationAnchors and IntegrateData with default parameters. Principal components (PCs) of the integrated and scaled data were computed; and the first 31 PCs, which accounted for >95% variance, were used in clustering cells. Cell clustering was performed using FindNeighbors and FindClusters with default parameters. Marker genes were identified in each cluster using FindMarkers in Seurat. Marker genes on one cluster must (1) be present in >20% cells in the cluster; (2) the log(fold change) between expression in the cluster and other clusters must be >0.25; (3) the rank sum test p‐value (Bonferroni‐adjusted) between cells in the cluster and cells in other clusters <0.05.

### 4.14 Meta‐analysis of bulk microglia RNAseq datasets

We obtained sets of co‐expression genes, that is, WGCNA modules from our own data and identified several modules whose eigengenes were correlated with age/sex/APOE. To check whether these correlations hold for external datasets, we downloaded raw reads in FASTQ format for three external bulk microglia datasets listed in Table S17. Reads were mapped to human genome build hg38 and were counted and normalized in the same fashion as described previously. For each WGCNA module, we identified the central genes (i.e., genes whose Pearson correlation with module eigengene > 0.75). Using these central genes, module eigengenes were calculated in all datasets—ours and three external ones. We correlated module eigengenes with traits. Finally, we performed meta‐analysis to combine correlations from multiple datasets using metacor function (random effect model) in R meta package.

### 4.15 Meta‐analysis of single‐cell RNAseq datasets

Raw single‐cell RNAseq data from Olah et al. (2020) was downloaded through Synapse (syn21438358) and processed through Cell Ranger. Quality control was performed as described prior to integration with our samples using Seurat v4.0.4. Integration was performed to combine the datasets by individual rather than sample. A total of 9 samples and 15,819 cells were retained from the Olah data. Cells were annotated to include dataset of origin. Hypergeometric tests were performed to determine the enrichment of our microglial signature within each integrated cluster. Additionally, we also looked at overlap between integrated cluster genes and marker genes from our original single‐cell dataset. To identify where our original cells localized within the integrated dataset, we mapped the original cell IDs to the integrated clusters and calculated percentage overlap.

## CONFLICT OF INTEREST

None.

## AUTHOR CONTRIBUTIONS

TP and NET wrote the manuscript; NET and MA designed the study; TP, XW, and ZQ performed data analysis; JC consulted on statistical methods; TP, TPC, XW, YM, and RMA generated tables and figures; EM, CAG, SG, KC, RW, HGC, and AQH provided neurosurgical tissue samples; TP, TPC, LJLT, SJL, SL, FQTN, CCGH, KGM, and TN performed experimental procedures from blood and tissue samples. All authors read the manuscript and provided input and consultation. NET oversaw the study and provided direction, funding, and resources.

## Supporting information

Fig S1‐S10

Table S1‐S20

## Data Availability

The data in this manuscript are available via the AD Knowledge Portal (https://adknowledgeportal.synapse.org). The AD Knowledge Portal is a platform for accessing data, analyses and tools generated by the Accelerating Medicines Partnership (AMP‐AD) Target Discovery Program and other National Institute on Aging (NIA)‐supported programs to enable open‐science practices and accelerate translational learning. The data, analyses, and tools are shared early in the research cycle without a publication embargo on secondary use. Data are available for general research use according to the following requirements for data access and data attribution (https://adknowledgeportal.synapse.org/DataAccess/Instructions). The raw data files are available through Synapse (https://www.synapse.org/#!Synapse:syn28450881.3/datasets/). **Table:** 

Dataset	Data Type	Description	SynapseID	DoD
Mayo RNAseq TCX	Metadata	Individual human and RNAseq	syn5550404	na
Mayo RNAseq CER	Metadata	Individual human and RNAseq	syn5550404	na
Mayo RNAseq TCX	RNASeq Expression	Consensus processed RNASeq raw counts	syn8690799	10/2/2019
Mayo RNAseq CER	RNASeq Expression	Consensus processed RNASeq raw counts	syn8690904	10/2/2019
ROSMAP	Metadata	ID Key	syn3382527	10/2/2019
ROSMAP	Metadata	Individual human	syn3191087	10/2/2019
ROSMAP	Metadata	Assay RNAseq	syn21088596	1/2/2020
ROSMAP	RNASeq Expression	Consensus processed RNASeq raw counts	syn8691134	10/2/2019
MSBB	Metadata	Individual human	syn6101474	11/22/2019
MSBB	Metadata	Assay RNAseq	syn6100548	10/2/2019
MSBB	RNASeq Expression	Consensus processed RNASeq raw counts	syn8691099	10/2/2019 **Data from AD knowledge portal utilized in this study.** DoD = Date of download, “na” indicates data that were generated by study authors and shared within the AD knowledge portal. Data were obtained from the RNAseq Harmonization Study in the AD knowledge portal (DOI: https://doi.org/10.7303/syn9702085).
